# The systemic administration of multipotent mesenchymal stromal cells alters the inflammatory response in the lung during murine polymicrobial sepsis

**DOI:** 10.1007/s00441-026-04102-4

**Published:** 2026-10-31

**Authors:** Anna Kanewska, Ina Lackner, Marco Dechant, Anne Friedrich, Martina Herrmann, Markus Rojewski, Mario Perl, Jochen Preßmar, Hubert Schrezenmeier, Miriam Kalbitz

**Affiliations:** 1 Department of Trauma and Orthopedic Surgery, University Hospital Erlangen, Friedrich-Alexander-Universität Erlangen-Nürnberg, Erlangen, Germany; 2 Department of Traumatology, Hand, Plastic and Reconstructive Surgery, Center of Surgery, University of Ulm, Ulm, Germany; 3 Institute for Transfusion Medicine, University Medical Center Ulm, Ulm, Germany; 4 Institute for Clinical Transfusion Medicine and Immunogenetics Ulm, German Red Cross Blood Donation Service, Ulm, Germany

**Keywords:** CLP sepsis, ARDS, Lung injury, ALI, Immune therapy, Stem cell therapy

## Abstract

Sepsis is a life-threatening organ dysfunction and a major cause of acute respiratory distress syndrome (ARDS). Mesenchymal stromal cells (MSCs) have emerged as a potential immunomodulatory therapy, yet preclinical data remain inconsistent. This study investigated the early effects of human MSCs derived from different tissue sources—bone marrow (BMMSCs) and adipose tissue (ASCs)—on systemic and pulmonary inflammation in a murine model of polymicrobial sepsis. 60 male C57BL/6 mice underwent sham treatment, cecal ligation and puncture (CLP), or CLP followed by a single intravenous administration of BMMSCs or ASCs, manufactured according to GMP-compliant protocols. Analyses were performed 8, 24, and 72 h after CLP. Systemic inflammatory mediators were quantified by multiplex analysis, while pulmonary inflammation was assessed by mRNA and protein expression, histology, and immunofluorescence. CLP induced elevated systemic levels of pro-inflammatory mediators, which were not significantly reduced by MSC treatment. In lung tissue, however, MSC administration resulted in selective immunomodulatory effects. Expression of NLR family pyrin domain containing 3 (*Nlrp3)* was reduced in both MSC-treated groups, while ASC treatment additionally decreased pulmonary interleukin 6 (*Il6)* and complement 3a receptor (*C3ar)* mRNA expression at early time points. Protein levels of IL-1β, IL-6, and TNF-α were not significantly altered, although NLRP3 protein expression was reduced following ASC treatment. No significant differences in histological lung injury scores were observed between groups. In summary, MSC treatment induced modest, time-dependent attenuation of pulmonary inflammatory responses in polymicrobial sepsis without improving structural lung injury. No significant differences in pulmonary inflammatory gene expression, protein levels, or histological lung injury scores were observed between BMMSC- and ASC-treated groups, indicating that neither source demonstrated a clear immunomodulatory advantage over the other at the molecular endpoints assessed in this model.

**Supplementary Information:**

The online version contains supplementary material available at https://doi.org/10.1007/s00441-026-04102-4.

## Introduction

Sepsis is defined as a life-threatening organ dysfunction, caused by a dysregulated host response to infection (Singer et al. 2016), that commonly affects the pulmonary system, making it the most frequent underlying cause of ARDS (Bellani et al. 2016; Xu et al. 2023). Pulmonary involvement in septic patients leads to hypoxemia and pulmonary dysfunction (Lelubre and Vincent 2018). At up to 40%, sepsis-induced ARDS has a higher mortality rate than ARDS with a non-septic etiology (Bellani et al. 2016; Englert et al. 2019; Li et al. 2023). Histologically, ARDS is characterized by diffuse alveolar injury driven by endothelial dysfunction and inflammation (Ferguson et al. 2012), with variable findings that may include intraalveolar edema, inflammatory cell infiltration, hyaline membrane formation, and alveolar hemorrhage, ultimately impairing gas exchange and leading to severe hypoxemia (Hu et al. 2020; Matthay et al. 2024).

During sepsis, various pro-inflammatory mediators such as damage-associated molecular patterns (DAMPs), cytokines and complement activation products are systemically elevated. They correlate with the development of ARDS, its severity, and associated mortality (Abraham et al. 2000; Parsons et al. 2005; Hotchkiss et al. 2016; Swaroopa et al. 2016; Yehya et al. 2024).

Management of sepsis is multifaceted and includes early hemodynamic stabilization, prompt antimicrobial therapy, and source control to eliminate the underlying infection (Gyawali et al. 2019; Evans et al. 2021; Guarino 2023; De Waele 2024).

A promising therapeutic approach would be to directly target the inflammatory response during sepsis to ameliorate the damage and improve the overall outcome of septic patients (Gotts and Matthay 2016; Hollis et al. 2025). In context ARDS, glucocorticoids have been shown to improve pulmonary physiology: glucocorticoids improve lung physiology, though effects on survival are context-dependent (Tongyoo et al. 2016; Liu et al. 2025). Beyond pharmacotherapeutic suppression of the immuno-inflammatory response, there is increasing evidence that mesenchymal stromal cell (MSC)-based therapies are a promising approach for host inflammatory disorders such as sepsis or ARDS (Matthay et al. 2010; Kusadasi and Groeneveld 2013).

Experimental studies have demonstrated early beneficial effects of stem cell therapy in sepsis models. Intravenous administration of murine bone marrow-derived MSCs (BMMSCs) in a CLP model of sepsis significantly improved organ function, as evidenced by reduced serum creatinine levels and enhanced survival (Luo et al. 2014). Moreover, reduced plasma levels of pro-inflammatory cytokines (Németh et al. 2009; Mei et al. 2010; Luo et al. 2014), diminished alveolar inflammatory cells and cytokines (Németh et al. 2009; Mei et al. 2010), were observed.

The beneficial effects of MSCs were primarily mediated via paracrine mechanisms, including suppression of TNF-α production of macrophages via interleukin-1 receptor antagonist protein (IL-1ra) (Ortiz, DuTreil et al. 2007) and enhancement of macrophage production of the anti-inflammatory cytokine IL-10 via prostaglandin E2 (PGE2) (Németh et al. 2009). These findings suggest that macrophages may be a potential key target of MSC-based therapies. In addition, MSCs have been shown to influence macrophage cytokine production and induce a polarization to anti-inflammatory M2-type macrophages (Selleri et al. 2016; Chen et al. 2021; Xing et al. 2023).

MSCs can be isolated from a variety of tissue sources, including bone marrow, adipose tissue and umbilical cord tissue (Kern et al. 2006, Shang et al. 2021). However, it remains controversial whether the tissue origin of MSCs influences their immunomodulatory potential (Bochev et al. 2008; Ivanova-Todorova et al. 2012; Elman et al. 2014; Ou et al. 2016). As a result, MSC-based therapies for sepsis have yielded inconsistent results, and the optimal tissue source for MSC isolation, particularly for clinical application, continues to be debated.

Previous experimental studies have demonstrated considerable differences in the immunomodulatory potential of MSCs, with variable effects on macrophages, T-cell, and B-cell proliferation, of which appeared to depend on the tissue source as well as origin of donor (Noël et al. 2008; Zhukareva et al. 2010; Pachón-Peña et al. 2011; Ménard and Tarte 2013; Bassi et al. 2015; Manferdini et al. 2020). In the present study, human BMMSCs and adipose tissue-derived MSCs (ASCs) were used. These cells were manufactured according to established GMP-compliant protocols that have already been applied in multiple clinical trials, where they demonstrated efficacy in improving bone consolidation (EudraCT No.: 2011–005441-13; EudraCT No.: 2012–002010-39, EudraCT No. 2012–003139-50). Xenogeneic administration of human MSCs in mouse models has been used in the past, without, to our knowledge, a description of the graft-versus-host response (Ramalho, Almeida et al. 2018; Sanchez-Diaz et al. 2021). However, the effect of these MSCs during sepsis, and in particular their immunomodulatory potential on the systemic and local pulmonal inflammatory response, has not been described yet. Therefore, the aim of this study was to investigate the development of ARDS on the lungs of mice subjected to CLP sepsis and assess whether inflammation can be ameliorated by the treatment of human MSCs from different tissue sources (bone marrow vs. adipose tissue).

## Materials and methods

### Manufacturing of BMMSCs and ASCs

BMMSCs and ASCs were manufactured according to good manufacturing practicing rules (GMP certificate number DE_BW_01_GMP_2020_0042) using aseptic procedures and disposable sterile single-use supplies for all product contact steps. Cell culture was performed as described for the manufacturing of clinical therapeutical products in the cell engineering unit quality system using the same or adapted standard operating procedures as previously described (Fekete et al. 2012). The following methodology of handling BMMSCs and ASCs and applying them to an animal model was performed in our previously published work in the exact same manner (Kanewska et al. 2025).

BMMSCs were generated from a 25-ml to 35-ml iliac crest bone marrow aspirate from healthy volunteer bone marrow donors (two male donors aged 23 and 27 years) after obtaining consent (accessed 30/07/2012, 17/09/2012, approved by Ethics Committee of the University of Ulm, Germany).

BMMSC isolation and expansion were performed according to the previously described standard operating procedures (Fekete et al. 2012; Rojewskiet al. 2019). Briefly, bone marrow cells were seeded at a density of 50,000 white blood cells (WBC) per cm^2^ on 1- or 2-chamber CellSTACKs (Corning, USA) in alphaMEM (Lonza, Switzerland), supplemented with 5% platelet lysate (PL) and 1 I.U. heparin/ml final concentration (PL from IKT Ulm, Germany) for isolation and first expansion step and with 8% PL and 1 I.U. heparin/ml final concentration for higher passages. The product consists of fresh bone marrow MSCs, expressing the identity markers CD90, CD73, and CD105, and lacking expression of impurity markers CD3, CD14, CD34, CD45, and HLA class II, with a 90% viability rate (Fekete et al. 2012). Such BMMSCs of passage 1 produced in this GMP-compliant manner have already been used in clinical trials as described previously and no serious adverse events were reported which were attributed to the BMMSC treatment (Gjerde et al. 2018; Gómez-Barrena et al. 2019; Gómez-Barrena et al. 2021). Here, 1 × 10^6^ cells were conditioned as described below and filled in a syringe for injection into each animal.

ASCs were generated from approximately 60 g of subcutaneous human adipose tissue from two healthy volunteers (provided without donor demographic data for data-protection reasons) via liposuction (accessed: 08/09/2015, 01/12/2015, approved by Ethics Committee of the University of Ulm, Germany) each. Aliquots of 20 to 25 ml of tissue were generated by digestion with GMP-grade collagenase (Nordmark, Germany), mixed with Dulbecco’s phosphate-buffered saline without Ca^2+^/Mg^2^ (DPBS) and centrifuged at 340 g for 4 min. The upper (oil and mature adipocytes) and lower (blood and fluid) phases were discarded. The stroma vascular fraction (SVF) was obtained from the remaining tissue by collagenase digestion (± 14 PZ-units of collagenase/10 g of pure adipose tissue; according to Wünsch (Wuensch and Heidrich 1963)) at 37 °C for 45 min. The digestion was stopped by the addition of alphaMEM, supplemented with 5% PL and 1 I.U./ml heparin. After homogenization of the solution by pipetting, the suspension was passed through a 100-μm cell strainer. The SVF was collected by centrifugation (20 °C, 10 min, 600 × g) and resuspended in media. SVF was seeded at a density of 4000 cells/cm^2^ in 2-chamber CellSTACKs in alphaMEM, supplemented with 5% PL and 1 I.U. heparin/ml. Media was exchanged after 4 and 6 days, and ASC of passage 0 were harvested after 8 days using TrypZean as detachment reagent. The product consist of fresh adipose-derived mesenchymal stromal cells (ASC), expressing the identity markers CD13, CD90, CD73, and CD105, and lacking expression of impurity markers CD3, CD14, CD34, CD45, and HLA class II, with a 90% viability rate (Fekete et al. 2012). ASCs of passage 1 produced according to this GMP-compliant protocol have already been used in the clinical trial ADIPOA2 (EudraCT No.: 2015–002125-19) for the treatment of mild to moderate arthritis (Kallgren-Lawrence-Scale grade 2 or 3) (manuscript in preparation). Further passaging for this study was performed by seeding the ASC on 2-chamber CellSTACKs at a density of 2000 cells/cm^2^ for passage 1 and 1000 to 2000 cells for passages ≥ 1 in alphaMEM, supplemented with 5% PL and 1 I.U. heparin/ml for another 4–6 days.

### Preparation of BMMSC and ASC injection

BMMSCs and ASCs were prepared for injection as previously published by our group (Kanewska et al. 2025). In brief, MSCs of passages ≤ 3 were detached using TrypZean, counted and incubated with 5000 I.U. heparin/ml at 20 °C for 30 min, rinsed once with DPBS and centrifuged at 400*xg* at 20 °C for 5 min prior to application. High-dose heparin pre-incubation was used to reduce MSC aggregate formation and to mitigate the risk of pulmonary microembolism following intravenous administration.

### Animal experiments

For the animal experiments, 60 male C57BL/6 mice (Jackson Laboratory) aged between 10 and 12 weeks and a weight between 25 and 30 g were used. All animal experiments were approved by the local animal welfare committee (Regierungspräsidium Tübingen, no. 1255) and followed international regulations for laboratory animal welfare and handling (ARRIVE guidelines for animal experiments). Littermate mice were housed in groups of up to five animals with free access to food and water ad libitum. Mice were randomly assigned to the following treatment groups: sham, CLP, CLP + BMMSC and CLP + ASC with *n* = 5 animals in each group. Animals were sacrificed at 8, 24, and 72 h after CLP. The time points were selected to capture the early pulmonary inflammatory response (8 h and 24 h) as well as a later read-out of lung injury resolution and possible structural changes (72 h).

### Surgical procedure

All animals were under general anesthesia and analgesia for surgical treatment. Anesthesia was induced by intraperitoneal (i.p.) administration of a mixture of 12.5 mg/ml (80–100 mg per kg of body weight) ketamine and 2.5 mg/ml (5–15 mg per kg of body weight) xylazine. For analgesia, mice received 0.05 mg/kg buprenorphine subcutaneously (s.c.) during surgery and every 6 h. To induce polymicrobial sepsis, CLP procedure as described previously by Rittirsch et al. (2009) and in our previous study (Kanewska et al. 2025). Briefly, the abdominal region was shaved, and a midline incision was given. The cecum was located and ligated halfway between the ileocecal valve and the cecal apex. Eventually the cecum was punctured with a 22-G needle and minimally extruded. Bowels were relocated and the incision was closed in layers.

Each animal was applied 1 mL of 0.9% saline s.c. for fluid resuscitation. Sham animals also received analgesia, anesthesia, abdominal shaving, and fluid resuscitation but no surgical procedure. CLP animals were randomly assigned into 3 therapy arms as soon as surgical procedure and fluid resuscitation were completed: PBS, BMMSC and ASC. In addition to the two human MSC sources compared here (BMMSC and ASC, cultured under identical GMP-compliant conditions), the original experiment included two exploratory arms using murine-derived MSCs and human BMMSCs cultured in fetal calf serum-containing medium. As these arms addressed different questions (species of origin and culture medium) and fell outside the scope of the present source comparison, they were not included in the analyses reported here. Corresponding lanes are present on the uncropped Western blots provided as Supplementary Figures, where they are indicated as (–), but were excluded from quantification.

Animals from the BMMSC group received 1 × 10^6^ BMMSCs, animals from the ASC group received 1 × 10^6^ ASCs, each resuspended in 250 µl DPBS via 1 ml syringes. Sham mice received an equivalent amount (250 µl) of sterile phosphate buffered saline (PBS). Each therapy was applied intravenously (i.v.) into the tail vein using a 30-G cannula. Subsequently, mice were allowed to move freely with access to food and water ad libitum. The observation periods were 8 h (*n* = 20), 24 h (*n* = 20), and 72 h (*n* = 20), during which the animals were closely monitored and received 0.05 mg/kg buprenorphine s.c. every 6 h for adequate pain medication.

### Sample collection

Mice were euthanized after the end of each observation period, 8, 24, and 72 h. Blood was taken directly from the heart immediately after euthanasia. Plasma samples were collected after centrifugation for 5 min (800 × *g*, 4 °C) and a second centrifugation step for 2 min (13,000 × *g*, 4 °C). The plasma samples were stored at − 80 °C until analysis.

Samples of lung tissue were taken from all mice and half of it either quick-frozen by liquid nitrogen or fixed in 4% paraformaldehyde for 48 h.

### Multiplex analysis

For determination of systemic inflammatory mediators, the plasma from mice was analyzed by using the Bio-Plex Pro Mouse Chemokine Assay (BIO-RAD, Hercules, CA, USA) and the Bio-Plex 200 (Luminex 200) Instrument and Software system (BIO-RAD, Hercules, CA, USA). The plasma was analyzed for GM-CSF, IL-6, KC/CXCL1, MCP1/CCL2, and IP-10/CXCL 10. All procedures were performed according to the manufacturer’s instructions.

### RNA isolation and RT-qPCR

Tissue from lung was homogenized with 0.5 ml Precellys® Lysing Kit 1.4 mm ceramic beads and Precellys®24 Tissue Homogenizer (Bertin Technologies, Montigny-le-Bretonneux, France), and RNA was isolated using AllPrep® DNA/RNA/miRNA Universal Kit (Qiagen, Hilden, Germany). The respective RNA samples were reverse transcribed into cDNA using SuperScript™ IV VILO™ MasterMix with ezDNAse (Invitrogen, Carlsbad, CA, USA). For quantitative PCR, the PowerUp™ SYBR™ Green Master Mix (Applied Biosystems, Waltham, MA, USA) and the QuantStudio3 system (Applied Biosystems, Waltham, MA, USA) were utilized. All procedures were performed according to the manufacturer’s instructions. Quantitative mRNA expression of murine *C3ar*, C5a receptor (*C5ar1*), *Il1b*, *Il6*, *Nlrp3*, *Tlr*2, *Tlr9,* and *Tnf*α was examined. Glyceraldehyde-3-phosphate dehydrogenase (*Gapdh*) was used as reference gene, assuming stable Gapdh expression across the experimental groups. A formal reference-gene stability analysis (e.g., geNorm/NormFinder) was not performed. Relative gene expression was calculated using the ΔΔCt-method. Gapdh was used for within-sample normalization and Sham-group as the calibrator. Results are presented as mean fold change.

### Protein isolation and gel electrophoresis

For protein isolation, lung tissue was homogenized using RIPA Lysis and Extraction Buffer (ThermoFisher Scientific, Waltham, MA, USA), including phenylmethanesulfonylfluoride (ThermoFisher Scientific, Waltham, MA, USA), protease Inhibitor Cocktail (Sigma-Aldrich, St. Louis, MO, USA), and sodium orthovanadate (Sigma-Aldrich, St. Louis, MO, USA). Lung tissue was homogenized by using the ULTRA TURRAX® T25 (IKA, Staufen, Germany). Total protein concentration of tissue homogenates was determined using Pierce™ BCA Protein Assay Kit (ThermoFisher Scientific, Waltham, MA, USA). For gel electrophoresis, 15 μg of total protein per lane, diluted in distilled water and Laemmli sample buffer and denatured at 96 °C for 7 min, with protein amounts equalized across all lanes based on BCA quantification, were loaded onto a 7–10% TGX Stain-Free™ FastCast™ acrylamide gel (BIO-RAD, Hercules, CA, USA).

### Western blot

After gel electrophoresis, the samples were transferred from 7 to 10% acrylamide gel onto Mini PVDF Membrane (BIO-RAD, Hercules, CA, USA) by using the Trans-Blot® Turbo™ Transfer System (BIO-RAD, Hercules, CA, USA). Membranes were blocked with either 5% dry milk or 5% bovine serum albumin (BSA) according to manufacturer’s instructions for 1.5 h at room temperature. After blockade, the membranes were incubated with the primary antibody against IL-1β (abcam, Cambridge, UK, ab205924, 1:1000; the pro-IL-1β precursor band at approximately 31 kDa was used for quantification), TNFα (abcam, Cambridge, UK, ab6671, 1:500), IL-6 (abcam, Cambridge, UK, ab208113, 1:1000), and NLRP3 (abcam, Cambridge, UK, [EPR23094-1] ab263899, 1:1000) overnight at 4 °C. After washing, membrane was incubated with anti-rabbit IgG HRP-linked antibody (CellSignaling Technology, Danvers, MA, USA) for 1 h at room temperature. Signal development was analyzed by ChemiDoc MP Imaging System (BIO-RAD, Hercules, CA, USA). Protein expression was quantified with the ImageLab Software (6.1.0. build 7, BIO-RAD, Hercules, CA, USA). Protein expression was normalized to the total lane protein. Results are expressed as mean pixel density.

### Immunofluorescence (IF)

For immunofluorescence analysis, formalin-fixed and paraffin-embedded tissue sections of the lung were used. Lung tissue sections were dewaxed and rehydrated. Antigen unmasking was performed by boiling the tissue sections in 10 mM citrate buffer (pH 6) at 100 °C. Non-specific binding sides were blocked by 10% goat serum. Specific antigen binding was performed by incubating the tissue sections with the respective primary antibody for CD163 (primary labeled with AlexaFluor®488, Bioss, Woburn, MA, USA, bs-23127R-A488) overnight at 4 °C. Cell nuclei were counterstained with Hoechst33342 (ThermoFisher, Waltham, MA, USA). The sections were investigated by fluorescence microscopy using an Axio Imager M.2 microscope (Zeiss, Jena, Germany). For each animal, three distinct representative fields of view were examined at 40 × magnification. For quantification of immunofluorescent staining, the ZEN 3.4 software (Zeiss, Jena, Germany) was used. A fluorescence intensity threshold was defined on a reference CD163^+^ cell, and all signals above this threshold were measured. All images were acquired at identical size and magnification, and each picture was analyzed independently. For each image, a background region in the alveolar air space was selected for background subtraction.

For each picture a specific mean value of fluorescence intensity (IF) was calculated by the software. Fluorescence intensity was not normalized to tissue area. Results are presented as mean fluorescence intensity (IF).

### Hematoxylin & Eosin (H.E.) staining

Formalin-fixed and paraffin-embedded tissue sections from lung tissue were used. Tissue sections were dewaxed and rehydrated. Pulmonal tissue sections were stained with hematoxylin & eosin staining kit (Morphisto, Frankfurt am Main, Germany). For quantification of pulmonal damage, a specific lung injury score was defined as described elsewhere (Matute-Bello et al. 2011). For determination of the lung injury score, the H&E-stained lung sections were scored independently by two investigators who were blinded to the experimental groups for (1) edema, (2) hemorrhage, (3) neutrophil infiltration, and (4) hyaline membrane. In each section, three representative sections were selected and the mean of the subsumed score calculated.

### Statistical analysis

Data were analyzed using GraphPad Prism 10.1.2 (GraphPad Software, Inc., San Diego, CA, USA). Given the small sample sizes and biological variability, non-parametric statistical methods were applied. Group comparisons were performed using the Kruskal–Wallis test followed by Dunn’s multiple comparison test. Untreated CLP mice were used as the reference group to assess treatment effects within the septic condition. Additional comparisons to sham-operated animals were performed as secondary analyses to contextualize disease induction and baseline expression levels. All data are presented as individual data points with the mean and standard deviation (SD). *P* ≤ 0.05 was considered statistically significant.

## Results

### Systemic inflammation in CLP animals with selective MSC-mediated effects

**Fig. 1:**
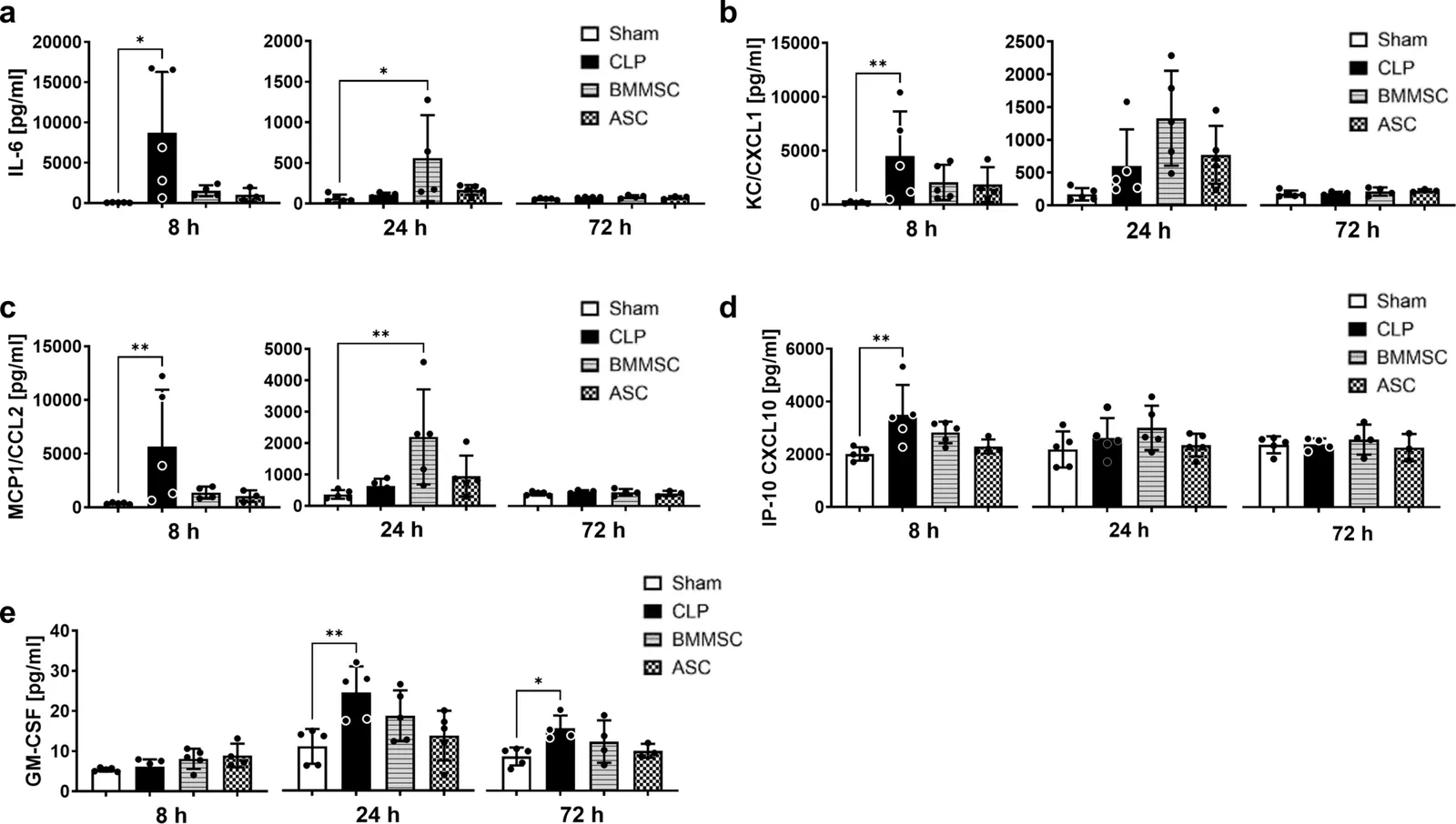
Systemic inflammation 8, 24, and 72 h following Cecal Ligation Puncture (CLP) sepsis. Male 10-12-week-old C57BL/6 mice received either sham treatment, CLP sepsis, or CLP sepsis with additional systemic application of human bone marrow-derived MSC (BMMSC) or of human adipose tissue-derived MSC (ASC). Blood plasma was analyzed 8, 24, and 72 h following sham, CLP, and CLP + BMMSC/ASC treatment. Systemic levels of interleukin-6 (IL-6) in pg/ml (**a**), keratinocyte chemoattractant (KC/CXCL1) in pg/ml (**b**), monocyte chemoattractant protein 1 (MCP1/CCL2) in pg/ml (**c**), C-X-C motif chemokine ligand 10/Interferon gamma-induced protein 10 (CXCL10/IP-10) in pg/ml (**d**), and granulocyte colonial stimulating factor (GM-CSF) in pg/ml (**e**). Data are presented as mean ± SD. *p* ≤ 0.05 was considered statistically significant. *p ≤ 0.05, **p ≤ 0.01

First, we analyzed the systemic levels of specific pro-inflammatory mediators in blood plasma 8, 24, and 72 h after CLP, CLP with BMMSC/ASC administration, and sham treatment. At 8 h, IL-6, KC/CXCL1, MCP1/CCL2, and IP-10/CXCL10 levels were systemically elevated in the CLP groups compared with sham, with the highest levels in the CLP group without MSC treatment (Fig. 1a–d). In contrast, GM-CSF levels were not significantly elevated in untreated CLP mice at this early point (Fig. 1e).

At 24 h, no increase in IL-6, KC/CXCL1, MCP1/CCL2, or IP-10/CXCL10 was detected in untreated CLP animals compared with sham (Fig. 1a–d), whereas GM-CSF levels were significantly elevated (Fig. 1e). In addition, BMMSC-treated animals exhibited increased systemic IL-6 and MCP1/CCL2 levels compared with sham (Fig. 1a). At 72 h, no significant group differences were observed for IL-6, KC/CXCL1, MCP1/CCL2, or IP-10/CXCL10 (Fig. 1a–d). GM-CSF was the only mediator showing a significant difference at this time point, with elevated levels in untreated CLP mice compared with sham; neither BMMSC nor ASC treatment significantly altered systemic GM-CSF levels relative to untreated CLP (Fig. 1e).

Administration of neither BMMSC nor ASC resulted in statistically significant changes in the expression levels of the respective pro-inflammatory mediators at any time point (Fig. 1a–c). Numerically lower mean values of IL-6, KC/CXCL1, and MCP1/CCL2 were observed at 8 h in MSC-treated groups compared to untreated CLP; however, these differences did not reach statistical significance and should be interpreted with caution given the small group sizes (*n* = 5).

### Lung tissue damage due to polymicrobial sepsis

**Fig. 2:**
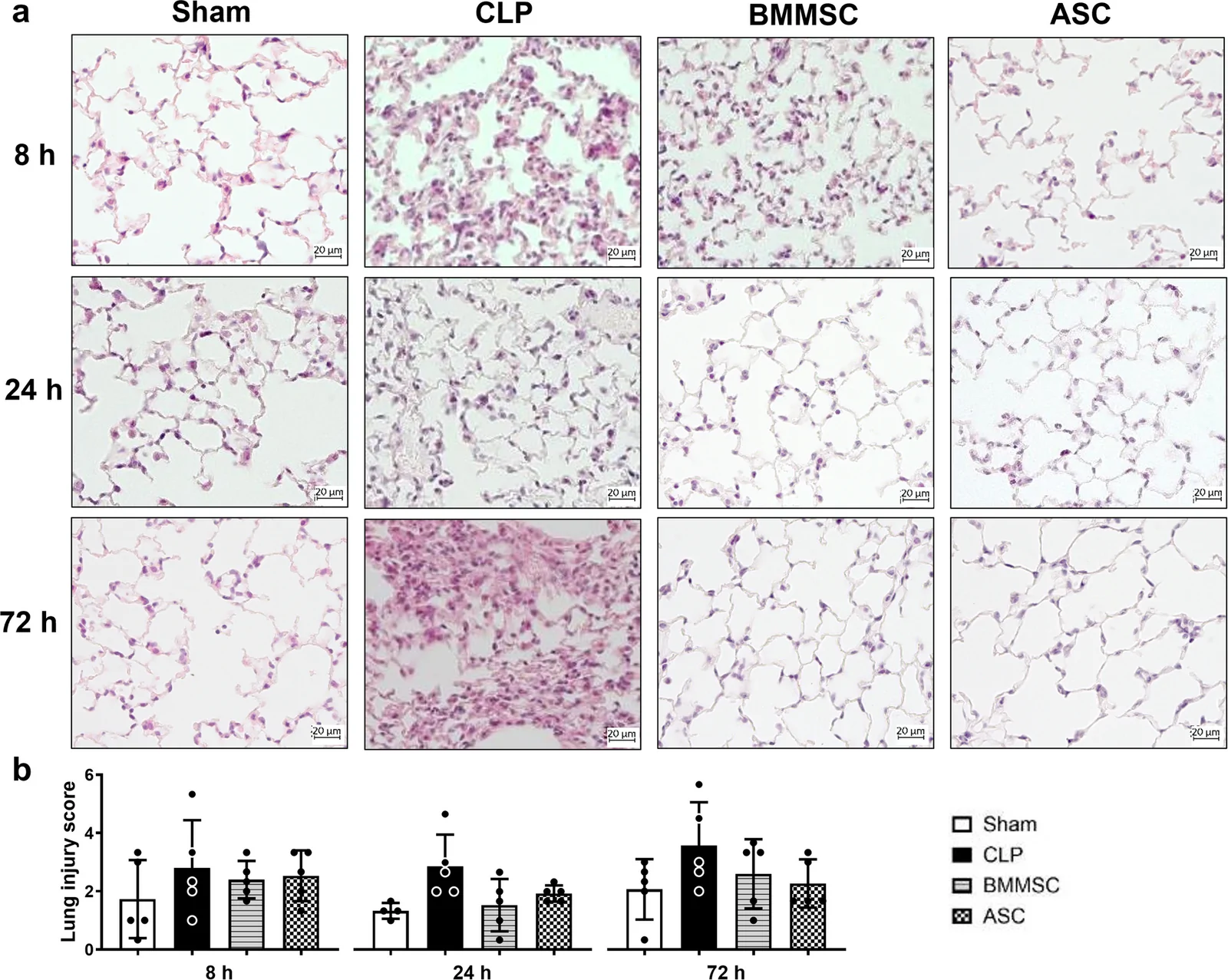
Histological lung injury 8, 24, and 72 h after Cecal Ligation Puncture (CLP) sepsis and macrophages infiltration. Male 10-12-week-old C57BL/6 mice received either sham treatment, CLP sepsis, or CLP sepsis with additional systemic application of human bone marrow-derived MSC (BMMSC) or of human adipose tissue-derived MSC (ASC). Representative hematoxylin and eosin (H&E)-stained lung Sects. 8 h, 24 h, and 72 h after CLP-surgery (**a**). Lung injury was quantified using a semi-quantitative injury score (**b**). No statistically significant differences were observed between groups at any time point. Data are shown as individual values with mean ± SD

To assess the extent of pulmonal damage, we evaluated pulmonal tissue Sects. 8, 24, and 72 h following CLP, CLP with BMMSC/ASC administration, and sham treatment (Fig. 2a), and determined a specific lung injury score for the respective groups at each observation time point (Fig. 2b). There were no statistically significant differences in lung injury scores between the groups at any time point (8 h, 24 h, or 72 h) after CLP. At 24 h, mean lung injury scores were numerically higher in the CLP group than in sham animals, but this difference was not statistically significant. At 72 h, lung injury scores likewise did not differ significantly between any of the groups (Fig. 2b).

### Expression of pro-inflammatory cytokines and NLRP3 inflammasome in lung tissue following CLP and MSC treatment

We analyzed local inflammation in lung tissue at 8, 24, and 72 h following sham treatment, CLP, and CLP with BMMSC or ASC administration, focusing on the expression of pro-inflammatory cytokines (IL-1β, TNFα, IL-6) and the NLRP3 inflammasome.

**Fig. 3:**
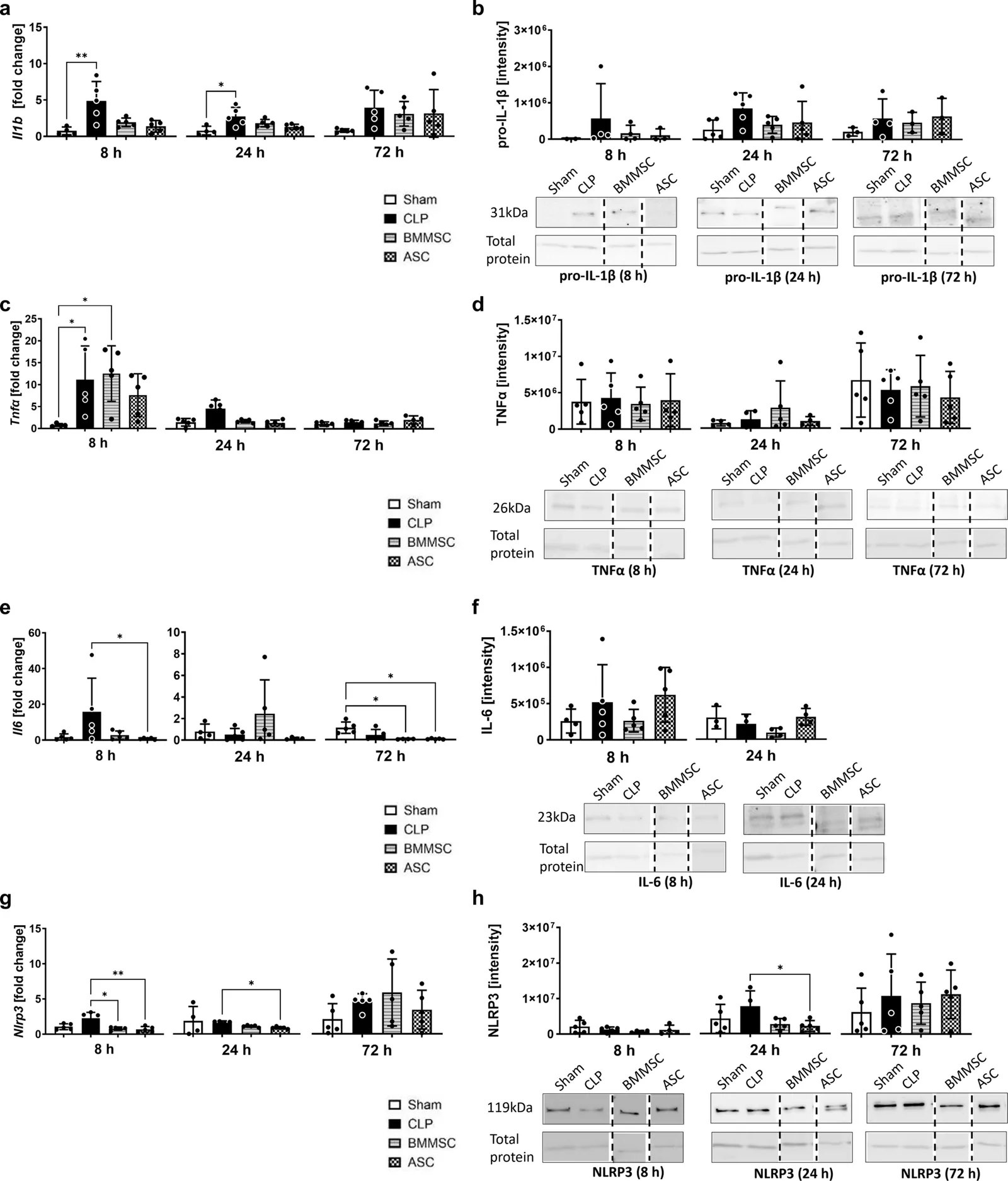
Local inflammation in septic lung tissue. Male 10-12-week-old C57BL/6 mice received either sham treatment, Cecal Ligation Puncture (CLP) sepsis, or CLP sepsis with additional systemic application of human bone marrow-derived MSC (BMMSC) or of human adipose tissue-derived MSC (ASC). Lung tissue was analyzed 8, 24, and 72 h following sham, CLP, and CLP + BMMSC/ASC treatment. Local interleukin-1β (*Il1b*) mRNA expression in fold change (**a**), local IL-1β protein expression in pixel intensity (**b**), local tumor necrosis factor (*Tnfa*) mRNA expression in fold change (**c**), local TNFa protein expression in pixel intensity (**d**), local interleukin-6 (*Il6*) mRNA expression in fold change (**e**), local IL-6 protein expression in pixel intensity (**f**), local NLR family pyrin domain containing 3 (*Nlrp3*) mRNA expression in fold change (**g**), and local NLRP3 protein expression in pixel density (**h**). Data are presented as mean ± SD. p ≤ 0.05 was considered statistically significant. *p ≤ 0.05, **p ≤ 0.01

At 8 h, *Il1b* gene expression was significantly elevated in CLP mice compared to sham (Fig. 3a), and this increase persisted at 24 h (Fig. 3a). However, pulmonary *Il1b* mRNA expression did not differ significantly between untreated CLP mice and BMMSC- or ASC-treated groups at either 8 h or 24 h after CLP (Fig. 3a). At the 72 h time point, *Il1b* mRNA expression likewise showed no significant differences between untreated CLP mice and BMMSC- or ASC-treated groups (Fig. 3a). Consistent with the transcriptional data, IL-1β protein levels in lung tissue were not significantly altered by BMMSC or ASC treatment compared to untreated CLP mice at any time point (Fig. 3b). IL-1β protein was assessed by immunoblotting and quantified at the 31 kDa pro-IL-1β band. No significant differences in pro-IL-1β levels were observed between untreated CLP mice and BMMSC- or ASC-treated groups at any time point.

*Tnfa* mRNA expression appeared elevated across all CLP groups at 8 h, reaching statistical significance in both, CLP and BMMSC compared to sham (Fig. 3c). At 24 h, pulmonary Tnfα mRNA expression did not differ significantly between untreated CLP mice and BMMSC- or ASC-treated groups (Fig. 3c). Consistent with the transcriptional findings, TNFα protein expression in lung tissue did not differ significantly between any of the experimental groups (Fig. 3D). At 72 h, neither *Tnfa* mRNA nor TNFα protein expression differed significantly between untreated CLP mice and BMMSC- or ASC-treated groups (Fig. 3c, d).

Pulmonary *Il6* mRNA expression did not differ significantly between untreated CLP and sham animals at either 8 h or 24 h (Fig. 3e). While ASC treatment significantly reduced *Il-6* mRNA expression at 8 h compared to untreated CLP, changes after BMMSC treatment were not significant. At 24 h, *Il6* mRNA expression did not differ significantly between any of the groups, with overall lower expression levels across all conditions (Fig. 3e). IL-6 protein levels also showed no significant differences between groups (Fig. 3f). At 72 h, both BMMSC- and ASC-treated mice showed significantly lower *Il6* mRNA fold-changes compared to sham; however, no significant difference was observed relative to untreated CLP mice (Fig. 3e), indicating that the addition of BMMSC or ASC to CLP did not significantly alter pulmonary *Il6* expression. At the protein level, no specific IL-6 band was detectable at the expected molecular weight of approximately 23 kDa at 72 h, despite comparable total protein loading across lanes; IL-6 protein was therefore considered below the limit of detection at this time point (Supplemental Figure).

*Nlrp3* mRNA expression did not differ significantly between untreated CLP and sham animals at either 8 h or 24 h (Fig. 3g). At 8 h, both BMMSC- and ASC showed significantly reduced *Nlrp3* mRNA expression compared to CLP. At 24 h, *Nlrp3* mRNA expression was significantly lower only in ASC-treated CLP mice compared to untreated CLP, whereas no significant difference was observed following BMMSC treatment.

Consistent with the mRNA findings, NLRP3 protein expression did not differ significantly between CLP and sham animals at either time point. However, ASC treatment resulted in significantly reduced NLRP3 protein expression compared to untreated CLP at 24 h, while no significant differences were observed following BMMSC treatment. At 72 h, neither *Nlrp3* mRNA nor NLRP3 protein expression differed significantly between untreated CLP mice and BMMSC- or ASC-treated groups (Fig. 3g, h).

### Complement activation and TLR signaling in lung tissue following CLP and MSC treatment

**Fig. 4:**
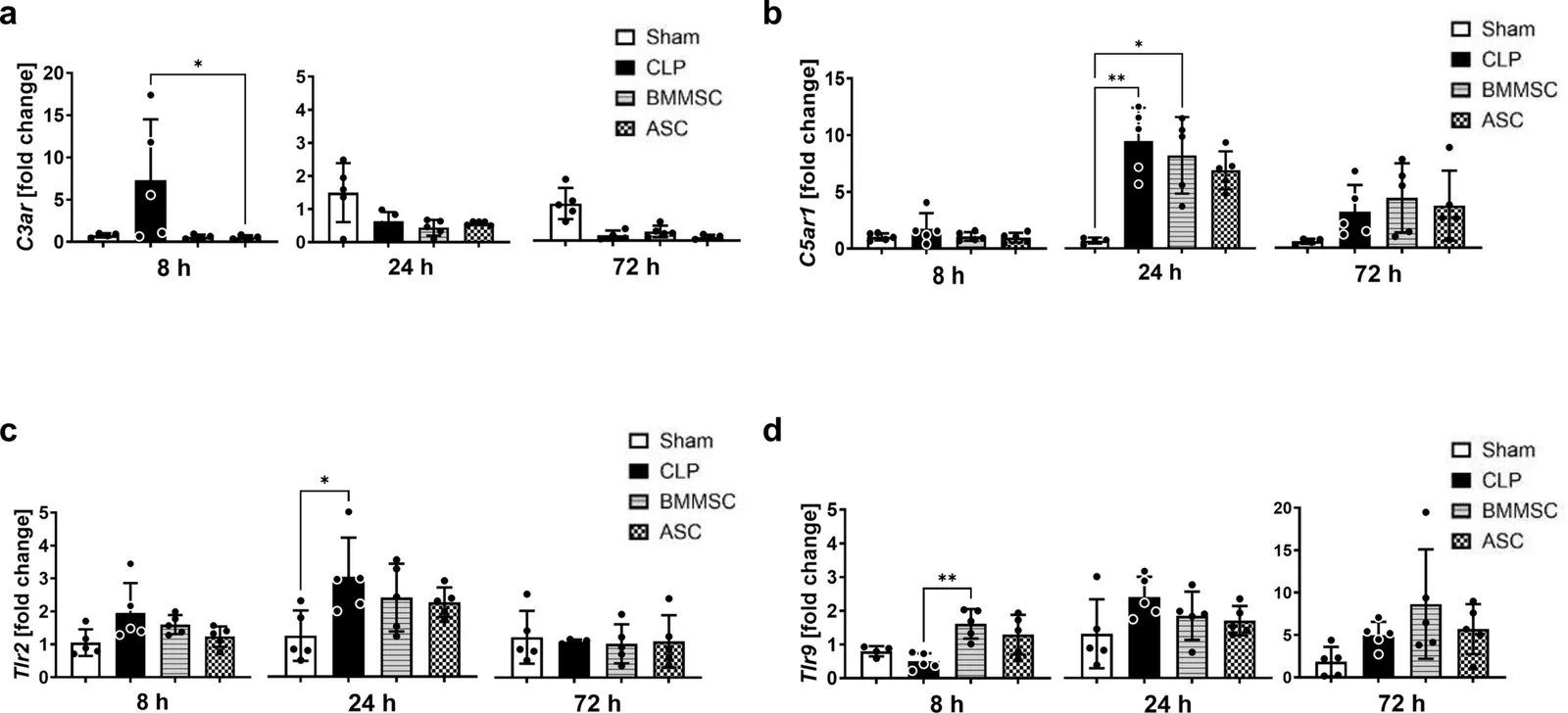
Local inflammation in lung tissue. Complement activation and toll-like receptor (TLR) signaling. Male 10-12-week-old C57BL/6 mice received either sham treatment, CLP sepsis, or CLP sepsis with additional systemic application of human bone marrow-derived MSC (BMMSC) or of human adipose tissue-derived MSC (ASC). Lung tissue was analyzed 8, 24, and 72 h following sham, CLP, and CLP + BMMSC/ASC treatment. Local C3a receptor (*C3ar*) mRNA expression in fold change (**a**), local C5a receptor 1 (*C5ar1*) mRNA expression in fold change (**b**), local toll-like receptor 2 (*Tlr2*) mRNA expression in fold change (**c**), and local *Tlr9* mRNA expression in fold change (**d**). Data are presented as mean ± SD. *p* ≤ 0.05 was considered statistically significant. *p ≤ 0.05, **p ≤ 0.01

We investigated complement activation (*C3ar*, *C5ar1*) and TLR signaling (*Tlr2*, *Tlr9*) in lung tissue at 8, 24, and 72 h following sham treatment, CLP, and CLP with BMMSC or ASC administration. At 8 h, *C3ar* mRNA expression did not differ significantly between CLP and sham (Fig. 4a). However, ASC treatment resulted in a significant reduction of *C3ar* mRNA expression compared to untreated CLP at this time point, whereas BMMSC treatment showed no significant effect (Fig. 4a). At 24 h, no significant differences in C3ar expression were observed between any of the groups (Fig. 4a). At 72 h, *C3ar* mRNA expression showed no significant differences between any of the groups (Fig. 4a).

No significant differences in *C5ar1* mRNA expression were detected between groups at 8 h (Fig. 4b). At 24 h, *C5ar1* mRNA expression was significantly elevated in CLP as well as BMMSC compared to sham, while ASC induced no significant effect. Neither MSC groups showed significant differences compared to CLP (Fig. 4b). At 72 h, *C5ar1* mRNA expression showed no significant differences between any of the groups (Fig. 4b).

Regarding TLR, *Tlr2* mRNA expression showed no significant differences between sham and any CLP group at 8 h (Fig. 4c) but was significantly elevated in untreated CLP compared to sham at 24 h (Fig. 4c). BMMSC and ASC treatment did not significantly alter *Tlr2* expression compared to untreated CLP at either time point (Fig. 4c). At 72 h, *Tlr2* mRNA expression showed no significant differences between any of the groups (Fig. 4c).

Moreover, no significant differences in *Tlr9* mRNA expression were observed between sham and CLP groups at either 8 or 24 h (Fig. 4d). However, BMMSC treatment significantly increased *Tlr9* expression compared to untreated CLP mice at 8 h (Fig. 4d). At 72 h, *Tlr9* mRNA expression showed no significant differences between any of the groups (Fig. 4d).

### Macrophage presence in the lungs of mice with polymicrobial sepsis after 24 h

**Fig. 5:**
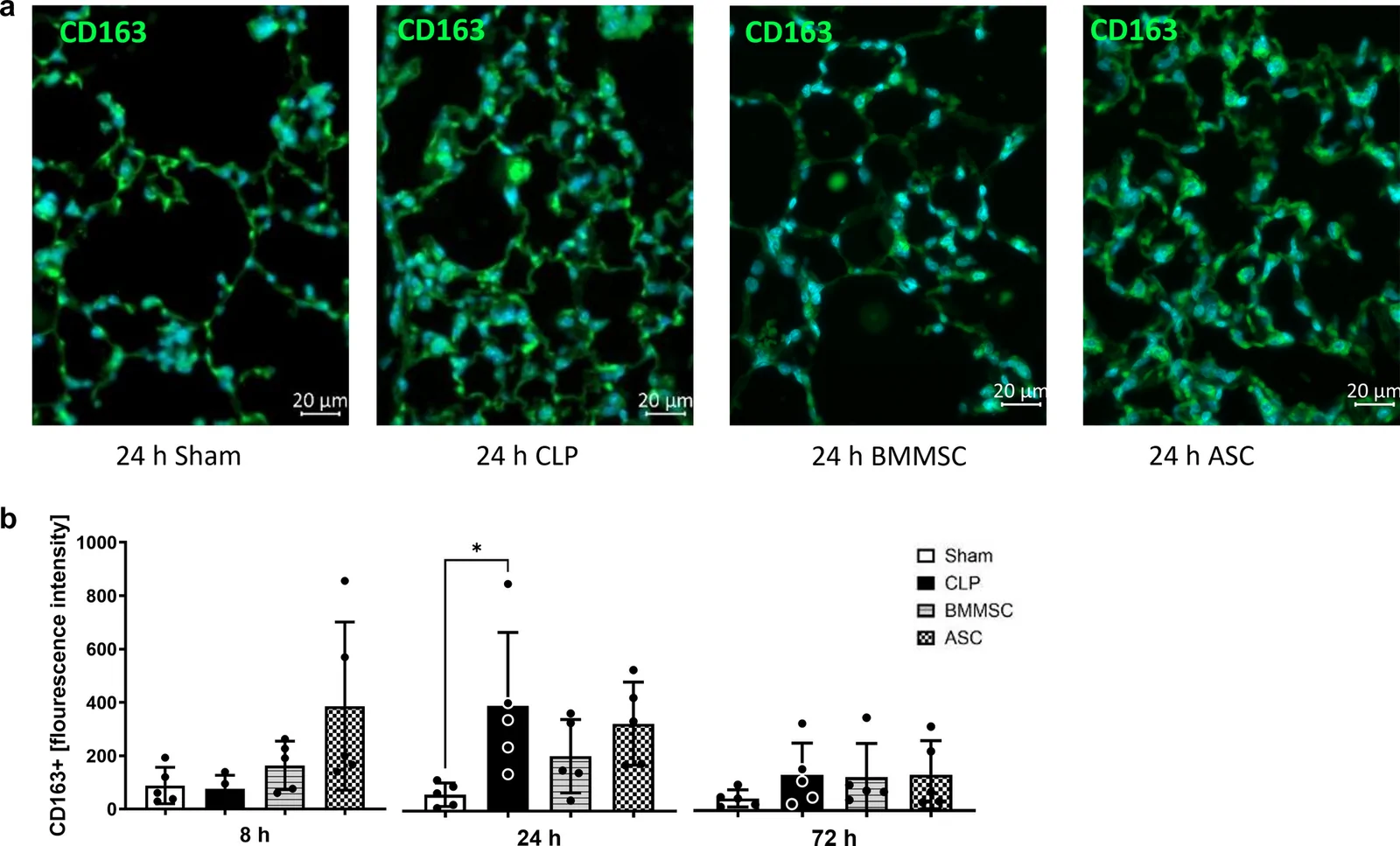
Infiltration of immune cells in lung tissue with higher CD163 expression in septic lung tissue. Male 10-12-week-old C57BL/6 mice received either sham treatment, Cecal Ligation Puncture (CLP) sepsis, or CLP sepsis with additional systemic application of human bone marrow-derived MSC (BMMSC) or of human adipose tissue-derived MSC (ASC). Lung tissue was analyzed 8, 24, and 72 h following sham, CLP, and CLP + BMMSC/ASC treatment. CD163 immunofluorescence staining of sham, CLP, BMMSC, and ASC (**a**) and CD163 expression in fluorescence intensity (**b**). Data are presented as mean ± SD. *p* ≤ 0.05 was considered statistically significant. *p ≤ 0.05

In addition to cytokine expression, macrophage activation and polarization contribute to pulmonary inflammatory responses during sepsis. To assess macrophage-associated immune responses in lung tissue following CLP and BMMSC or ASC administration, we analyzed the expression of CD163, a scavenger receptor commonly used as a marker of macrophages with anti-inflammatory or resolution-associated phenotypes (Fig. 5).

At 8 h, no significant differences in CD163 expression were observed between any of the groups (Fig. 5b). At 24 h, CD163 expression was significantly increased in untreated CLP mice compared to sham controls. Neither BMMSC nor ASC treatment significantly altered CD163 expression relative to untreated CLP at this time point (Fig. 5b). At 72 h, no significant differences in CD163 expression were observed between any of the groups (Fig. 5b).

## Discussion

This study investigated the immunomodulatory effects of human bone marrow-derived and adipose tissue-derived MSCs in a murine model of polymicrobial sepsis and demonstrated a mild, time-dependent attenuation of pulmonary inflammatory responses.

In a broader pathophysiological context, systemic inflammation plays a critical role in the development of sepsis-associated organ dysfunction, particularly acute respiratory distress syndrome (ARDS). Sepsis-induced ARDS is not only associated with increased systemic levels of various pro-inflammatory mediators such as IL-6 or IL-8, but their levels also correlate with increased mortality in patients (Parsons et al. 2005; Swaroopa et al. 2016). Accordingly, in this study, the mice of the CLP group showed increased systemic levels of IL-6, KC and MCP1 after 8 h. This is consistent with previous in vitro and clinical studies (Kang et al. 2020) as well as animal studies in mice (Zhao et al. 2019), which also showed a correlation with the development of sepsis-associated ARDS.

In addition, mice receiving a single intravenous administration of BMMSCs, or ASCs showed a tendency for decreased systemic IL-6, KC and MCP1 levels 8 h after CLP. This observation may indicate a beneficial immunomodulatory effect of MSCs in attenuating the systemic pro-inflammatory response early in polymicrobial sepsis. Reduced levels of cytokine-induced neutrophil chemoattractant 1 (CINC-1) and IL-6 have been recently described during CLP sepsis after administration of human umbilical cord MSCs in rats (Gonzalez et al. 2020) and murine bone marrow MSCs in mice (Németh et al. 2009; Mei et al. 2010; Luo et al. 2014). This was further associated with improved survival and reduced pulmonal damage of the animals, particularly when administered within an effective therapeutic window (Mei et al. 2010; Gonzalez et al. 2020).

In line with these observations, our data indicates a modest attenuation of inflammatory mediators at early time points following direct MSC administration. However, this pattern was not sustained. At 24 h, BMMSC-treated mice exhibited significantly elevated systemic IL-6 and MCP1 levels compared with sham mice, whereas ASC treatment did not induce comparable late cytokine increases. Notably, untreated CLP mice did not display significant elevation of these mediators relative to sham controls at this time point. As systemically administered MSCs are known to undergo rapid clearance while exerting prolonged immunomodulatory effects through interactions with host immune cells (Eggenhofer et al. 2012; Masterson et al. 2019), the late cytokine elevation observed in BMMSC-treated animals may therefore reflect host immune responses rather than direct MSC-derived production.

The differential temporal patterns between BMMSC and ASC treatment may suggest source-dependent differences in host immune activation. Notably, ASC treatment has been shown to maintain sustained anti-inflammatory effects for up to 24 h in rodent (Sun et al. 2020) and 7 days in large animal sepsis models (Vélez-Pinto et al. 2023), while the mechanisms underlying these source-dependent differences remain unclear.

Besides systemic inflammation, the development of septic-induced lung injury is characterized by an exuberant local inflammatory response in the lung, involving recruitment of inflammatory cells and release of pro-inflammatory mediators that disrupt the alveolar-capillary barrier and contribute to acute lung injury and ARDS (Xu et al. 2024).

To assess the local pulmonal inflammatory response, we first analyzed the expression of the pro-inflammatory cytokines IL-6, IL-1β and TNFα as well as of the pattern recognition receptors (PRR) NLRP3, TLR2, and TLR9. In accordance with the systemic data, the pulmonary mRNA expression of *Il1b, Il6,* and *Nlrp3* significantly increased after 8 h in the CLP group but not in the BMMSC and ASC groups. Previous studies have demonstrated increased pulmonary IL-1β and IL-6 expression during CLP sepsis and their association with the development of lung injury (Meng et al. 2019; Alnfakh et al. 2022). These reductions in pro-inflammatory cytokine expression with MSC treatment are consistent with prior reports showing that murine BMMSCs downregulate pulmonary *Il6* and associated signaling pathway modulators including TNF-receptor-associated factor 6 (Traf6) and transcription factor c-Jun in CLP mice (Mei et al. 2010). However, IL-1β activity is primarily regulated post-translationally via inflammasome-dependent cleavage, and mRNA expression alone does not directly reflect bioactive cytokine levels. Therefore, these findings should be interpreted as changes in inflammatory priming rather than definitive evidence of altered IL-1β signaling activity.

Notably, in our previous study using the same CLP model with cardiac tissue analysis, BMMSCs predominantly modulated complement system expression while ASCs more strongly influenced *Il6* (Kanewska et al. 2025), suggesting that MSC immunomodulatory effects may be organ-specific.

The observed reductions in pulmonary *Nlrp3* expression following BMMSC and ASC administration at 8 h suggest modest modulation of innate immune activation within lung tissue. While pharmacological NLRP3 inhibition has been shown to ameliorate CLP-induced lung injury (Wang et al. 2015; Li et al. 2021), whether MSCs exert comparable effects on inflammasome activation remains to be demonstrated. Notably, these MSC-mediated effects were transient and did not translate into sustained structural protection, underscoring the limited and context-dependent nature of MSC immunomodulation in this model.

The lung-specific effects observed may relate to MSC biodistribution, as systemically administered MSCs become predominantly trapped in the lung (Barbash et al. 2003; Fischer et al. 2009; Eggenhofer et al. 2012). Interestingly, pulmonary *Tlr9* mRNA expression was significantly elevated in BMMSC-treated mice compared to untreated CLP at 8 h. This elevation may reflect host recognition of degrading MSCs, as cellular debris can act as damage-associated molecular patterns (DAMPs) activating TLR9 signaling. Pulmonary *Tlr2* expression was similarly elevated in untreated CLP mice, consistent with previous reports of TLR2 upregulation in septic lung tissue (Bakopoulos et al. 2017), though no significant differences were observed between MSC-treated and untreated groups.

Sepsis-induced lung injury is further associated with the immediate activation of the complement system. Pulmonary C3a generation and C3ar activation during CLP sepsis have been associated with the development of sepsis-induced lung injury (Li et al. 2022), while elevated C5ar1 levels have been associated with impaired survival (Riedemann et al. 2003; Ward 2010).

In the present study, pulmonary C3ar mRNA expression at 8 h showed a non-significant increase in untreated CLP compared to sham animals. ASC treatment significantly reduced C3ar expression relative to CLP, whereas BMMSC-treated mice showed lower mean expression that did not reach significance. These findings suggest possible modulatory effect of ASC on the C3a-C3aR axis. This aligns with previous reports showing that MSC-secreted exosomes alleviate immune dysregulation in acute respiratory failure by inhibiting complement activation (Loh et al. 2022) and that MSCs reduce C5ar1 expression following ischemia/reperfusion injury (Tang et al. 2018).

In contrast, pulmonary *C5ar1* expression was significantly elevated at 24 h in both untreated and BMMSC-treated CLP mice, with no MSC-mediated attenuation observed in either group. This divergence between C3ar and C5ar1 modulation may reflect distinct activation dynamics within the complement cascade, as C5a can be generated through extrinsic proteases independent of classical C3 convertase pathways (Huber-Lang et al. 2006; Foley et al. 2016). These findings suggest that *C3ar* expression may be more sensitive to upstream immunomodulatory interventions than *C5ar1*, and that MSC treatment only partially modulates complement activation in this model.

The present study demonstrated elevated systemic and pulmonary pro-inflammatory cytokine expression 8 h post-CLP, with selective, statistically significant reductions in specific pulmonary inflammatory markers following MSC administration, while systemic cytokine levels were not significantly altered. These findings are consistent with proposed MSC immunomodulatory mechanisms, including macrophage reprogramming and polarization toward anti-inflammatory M2-type macrophages expressing CD163 (Ortiz et al. 2007; Németh et al. 2009; Mei et al. 2010; Selleri et al. 2016; Chen et al. 2021; Luque-Campos et al. 2021; Xing et al. 2023). While CD163 expression showed a numerical increase in ASC-treated animals at 8 h, this did not reach statistical significance. By 24 h, CD163 was elevated across all CLP groups, limiting interpretation of MSC-specific effects. Further studies are needed to definitively demonstrate M1-to-M2 macrophage polarization following MSC treatment in this model.

These findings should be interpreted in light of several limitations. The experimental design did not include assessment of clinical symptoms or functional lung parameters comparable to those used in human ARDS. Histological lung injury and inflammatory markers were the primary outcome measures, and no differences in structural lung damage were observed between groups. Given the relatively mild lung injury in this model, alternative approaches such as intravenous LPS administration may be more appropriate for inducing robust histological lung damage. These considerations reflect the intentionally controlled, mechanistic design of the study, which was conceived as an exploratory head-to-head comparison of two GMP-manufactured MSC sources rather than as a model of clinical treatment. Three main limitations should be borne in mind when interpreting the findings. First, the model itself has limited translational fidelity: the uncontrolled CLP procedure was performed without antibiotics or extended fluid resuscitation, MSCs were administered immediately after induction, and no physiological pulmonary endpoints were assessed. In addition, the sham group did not undergo a sham laparotomy, so that the surgical component of the CLP procedure was not fully controlled. These features, together with the intravenous delivery of xenogeneic human MSCs, mean that the results should be read as hypothesis-generating for MSC immunomodulation rather than as direct evidence of therapeutic efficacy in established sepsis-associated ARDS. Second, the immunomodulatory effects were predominantly transcriptional, of modest magnitude, and largely resolved by 72 h, with limited concordance between mRNA and protein data; in particular, the reduced NLRP3 expression should not be over-interpreted, as downstream markers of inflammasome activation (Caspase-1, IL-18, ASC speck formation, GSDMD cleavage) were not assessed. Third, the small group sizes (*n* = 5) limit statistical power, so that the absence of significant differences should not be interpreted as equivalence, and inter-donor variability inherent to MSC products may have contributed to the variability observed. In addition, the CD163 immunofluorescence signal was not normalized to tissue area. Given the variable alveolar air space, this may contribute to differences in signal intensity between groups. Despite these constraints, the study provides a controlled, GMP-relevant comparison of bone marrow- and adipose-derived MSCs under identical conditions, and delineates specific, testable endpoints, most notably inflammasome processing and later structural outcomes, for adequately powered studies incorporating clinically relevant supportive care.

Both MSC types selectively reduced specific pulmonary inflammatory markers at early time points; ASC treatment more consistently reached statistical significance, while BMMSC effects were more variable with some markers elevated at later time points. These source-dependent differences suggest distinct immunomodulatory kinetics that may be influenced by timing and inflammatory context, though the small sample size limits definitive interpretation. Overall, MSC-mediated effects were modest and selective in this model, underscoring the context-dependent nature of MSC immunomodulation rather than a universally robust anti-inflammatory response.

Despite these modest effects and the limitations outlined above, MSC administration consistently modulated inflammatory responses in lung tissue, particularly at early time points following sepsis induction. These findings support a role for MSCs in early immunomodulation during sepsis-associated lung inflammation, while highlighting the need for future studies incorporating clinically relevant functional endpoints, larger sample sizes for improved statistical power, and complementary animal models with more robust lung injury to better assess translational relevance.

## Conclusion

In this murine model of polymicrobial sepsis, both bone marrow-derived and adipose tissue-derived MSCs exerted modest immunomodulatory effects without improving pulmonary injury scores or systemic outcome parameters. At the pulmonary level, MSC treatment was associated with reduced expression of inflammasome-related markers such as *Nlrp3*, while ASC treatment additionally attenuated *Il6* expression at early time points. However, these molecular effects were time-dependent, limited in magnitude, and did not translate into sustained systemic anti-inflammatory responses. Overall, the data indicates that MSC therapy confers only partial and context-dependent immunomodulation in sepsis, without demonstrating a clear immunomodulatory advantage of one MSC tissue source over the other at the molecular and histological endpoints assessed.

## Supplementary Information

Below is the link to the electronic supplementary material.

ESM 1 (DOCX 24.0 MB)

ESM 2 (DOCX 14.8 KB)

## Data Availability

The datasets analyzed during the current study are available from the corresponding author on reasonable request.

## Abbreviations

*PGE2*: Prostaglandin E2
*ASC*: Adipose Stromal Cells
*BMMSC*: Bone marrow-derived mesenchymal stromal cells
*CCL2*: CC-chemokine ligand-2
*CLP*: Cecal ligation puncture procedure
*C3ar*: Complement component 3a receptor
*C5ar1*: Complement component 5a receptor 1
*CXCL1*: C-X-C motif chemokine ligand 1
*DAMP*: Damage-associated molecular pattern
*ELISA*: Enzyme-Linked Immunosorbent Assay
*GM-CSF*: Granulocyte-macrophage colony-stimulating factor
*GMP*: Good manufacturing practice
*ICU*: Intensive Care Unit
*IL-1ra*: Interleukin-1 receptor antagonist protein
*Il-10*: Interleukin-10
*Il-1ß*: Interleukin-1β
*Il-6*: Interleukin-6
*i.p.*: Intraperitoneally
*IP-10*: Interferon gamma-induced protein 10
*KC*: Keratinocyte chemoattractant
*MCP1*: Monocyte chemoattractant protein-1
*MSC*: Mesenchymal stromal cells
*Nlrp3*: NOD-, LRR- and pyrin domain-containing protein 3
*PFA*: Paraformaldehyde
*PBS*: Phosphate buffered saline
*qPCR*: Quantitative reverse transcription polymerase chain reaction
*s.c.*: Subcutaneously
*Tlr2*: Toll-like receptor 2
*Tlr9*: Toll-like receptor 9
*Tnfa*: Tumor necrosis factor alpha
*TNF α*: Tumor necrosis factor α
