# Supplementary material for: The systemic administration of multipotent mesenchymal stromal cells alters the inflammatory response in the lung during murine polymicrobial sepsis
Source: Cell Tissue Res. 2026 Oct 31;406(1):1. doi: 10.1007/s00441-026-04102-4 (PMC13722731; doi:10.1007/s00441-026-04102-4)
Supplement: Supplementary file 1 [file 441_2026_4102_MOESM1_ESM.docx]

**A**

**B**

**C**

**D**

**Supplementary Figure 1. Full uncropped Western blots for all analyzed cytokines**

Complete, uncropped Western blot membranes corresponding to the representative blots shown in Figure 3. Panels show (**A**) IL-1β, (**B**) TNF-α, (**C**) IL-6 and (**D**) NLRP3. For each cytokine and each time point (8, 24 and 72 h), all four membranes are shown. For every membrane, the chemiluminescence signal for the target protein and the corresponding stain-free total-protein image (loading control) are shown. Target signal was normalized to total lane protein using the stain-free imaging system. Quantification was based on the following bands: pro-IL-1β at approximately 31 kDa (A), TNF-α at approximately 26 kDa (B), IL-6 at approximately 23 kDa (C) and NLRP3 at approximately 119 kDa (D). At 72 h, no specific IL-6 band was detectable at approximately 23 kDa despite comparable total-protein loading (C). Lanes are labelled by experimental group (Sham, CLP, BMMSC, ASC). Lanes marked (–) correspond to exploratory experimental arms (murine-derived MSCs and human BMMSCs cultured in fetal calf serum-containing medium) that fall outside the scope of the present source comparison and were excluded from quantification. Molecular weight markers (in kDa) are indicated.
