# Supplementary material for: The systemic administration of multipotent mesenchymal stromal cells alters the inflammatory response in the lung during murine polymicrobial sepsis
Source: Cell Tissue Res. 2026 Oct 31;406(1):1. doi: 10.1007/s00441-026-04102-4 (PMC13722731; doi:10.1007/s00441-026-04102-4)
Supplement: Supplementary file 2 [file 441_2026_4102_MOESM2_ESM.docx]

**SUPPLEMENTAL TABLE**

| **Primer (all murine)** | **forward sequence** | **Reverse sequence** |
| --- | --- | --- |
| C3a receptor (C3aR) | 5‘-CATCGAAACGTGAGAACCGC-3‘ | 5‘-CGGGCACACACATCACAAAG-3‘ |
| C5a receptor 1 (C5aR1) | 5‘-CCAGGACATGGACCCCATAG-3‘ | 5‘-ATGCCATCCGCAGGTATGTT-3‘ |
| glyceraldehyde-3-phosphate dehydrogenase (GAPDH) | 5‘-CTTCAACAGCAACTCCCACTCTTCC-3‘ | 5‘-GGTGGTCCAGGGTTTCTTACTCC-3‘ |
| interleukin (IL)-1β | 5‘-GCCACCTTTTGACAGTGATGAG-3‘ | 5‘-TGACAGCCCAGGTCAAAGGTT-3‘ |
| IL-6 | 5‘-CCCCAATTTCCAATGCTCTCC-3‘ | 5‘-GGATGGTCTTGGTCCTTAGCC-3‘ |
| NLR family pyrin domain containing 3 (NLRP3) | 5‘-GCTGCTCAGCTCTGACCTCT-3‘ | 5‘-AGGTGAGGCTGCAGTTGTCT-3‘ |
| toll-like receptor (TLR)2 | 5‘-GAAACCTCAGACAAAGCGTCA-3‘ | 5‘-ACAGCGTTTGCTGAAGAGGA-3‘ |
| TLR9 | 5‘-GAGAGACCCTGGTGTGGAAC-3‘ | 5‘-CCTTCGACGGAGAACCATGT-3‘ |
| tumor necrosis factor (TNF) | 5‘-AAGCTCCTCAGCGAGGACAG-3‘ | 5‘-TGGTTGGCTGCTTGCTTTTC-3‘ |
